# Abdominal obesity and associated factors among urban adults in Southwest Ethiopia: a community-based cross-sectional study

**DOI:** 10.11604/pamj.2024.47.47.34746

**Published:** 2024-02-06

**Authors:** Fitsum Endale, Aderajew Nigussie, Aiggan Tamene, Aklilu Habte, Dejene Ermias, Abera Beyamo, Tegegn Tadesse, Dawit Sulamo, Tefera Belachew

**Affiliations:** 1 School of Public Health, College of Medicine and Health Sciences, Wachemo University, Hosanna, Ethiopia,; 2 Department of Nutrition and Dietetics, Institute of Health Sciences, Jimma University, Jimma, Ethiopia

**Keywords:** Adult, abdominal obesity, Jimma town, Southwest Ethiopia

## Abstract

**Introduction:**

the obesity epidemic is growing faster in developing countries with no exception of Ethiopia. Currently, abdominal obesity is identified as a major risk factor for chronic diseases due to the accumulation of liable fat. However, despite the evidence of certain documented data, abdominal obesity has been on the rise in Ethiopia, especially in urban areas. Therefore, this study aimed to assess the prevalence and factors associated with abdominal obesity among adults in Jimma town, Southwest Ethiopia.

**Methods:**

a community-based cross-sectional study was employed on 845 adults selected using a multi-stage sampling technique. Data were collected using a pretested interviewer-administered questionnaire. Data were entered using Epi-data version 3.1 and exported to STATA version 14 for analysis. Simple linear regression was conducted to identify candidate variables. A multivariable linear regression model was fitted to identify factors associated with abdominal obesity. P-value<0.05 was used to declare statistical significance.

**Results:**

a total of 806 respondents participated in this study, making a response rate of 95.4%. The magnitude of abdominal obesity was found to be 24.6% (95% CI: 21.5, 27.5). Physical activity (β= -2.053; 95%CI: -3.353, -0.454), alcohol consumption (β=1.631; 95%CI: 0.176, 3.087), and age (β=0.319; 95%CI: 0.250, 0.389) were significantly associated with abdominal obesity.

**Conclusion:**

the magnitude of abdominal obesity among adults in the study area was high compared to previous studies. Alcohol drinking, being physically inactive, and age were predictors of abdominal obesity. There is a need for intervention for adults with physical inactivity and alcohol consumption to reduce abdominal obesity.

## Introduction

Globally, the prevalence of overweight and obesity has more than doubled between 1980 (4.8% for men and 7.9% for women) and 2016 (10% of men and 14% of women) [1]. The obesity epidemic is growing faster in developing countries [2]. Based on published reports, up to 50% of the African population who are living in urban areas are expected to be under obesity or overweight, and by 2025 the majority of obese individuals will be found in low and middle-income countries [3]. Even though it has variations, the prevalence of obesity in Africa ranges from as low as 0.6% in the Gambia to as high as 80.2% in Nauru [4]. Similarly, in Ethiopia, the prevalence of obesity has increased more than sixfold between 1990 and 2009 [5,6].

Though the method most commonly used in the determination of obesity is Body Mass Index (BMI), however, BMI does not provide enough information regarding the distribution of fat in the body [7]. Evidence has shown that fat distribution is an important marker for obesity compared to the total amount of fat alone and it was evidenced that waist circumference is the simplest anthropometric measurement to be used to determine both the description of obesity and for identifying the individuals who are at an increased risk for developing chronic diseases [8,9]. Abdominal obesity is associated with an increased risk of metabolic syndrome and Cardiovascular Diseases (CVD) due to liable fat [10]. Independent of BMI or total body fat, abdominal obesity is associated with increased risks of heart disease, stroke, diabetes, hypertension, gallstones, and some types of cancer [11,12]. World Health Organization (WHO) has classified individuals as abdominally obese, when a male's Waist Circumference (WC) is greater than or equal to 94 centimeters (cm) and a female's WC is greater than or equal to 80 cm [13].

The factors for the development of abdominal obesity are believed to be multi-causal and different studies have found that lifestyle and behavioral changes such as unhealthy diet, physical inactivity, smoking, and alcohol drinking are the major modifiable factors that are believed to be the major contributing factors for the global epidemic of obesity [14,15]. These behavioral risk factors along with obesity are responsible for a substantial increase in the prevalence of intermediate CVD risk factors, including hypertension and diabetes [16], which results in a double burden, especially in low and middle-income countries, with no exception of Ethiopia [17].

To avert this devastating problem more recently the Ethiopian government has given a growing recognition to the worrying trends in the magnitude of chronic diseases and their risk factors. As a result, the government included Non-communicable Diseases (NCDs) as one component of the National Nutrition Program (NNP) [18] with the following initiatives: promoting public awareness of healthy lifestyles, integrating prevention and control of lifestyle related diseases in the urban health extension program to enhance physical activities of the community. The Federal Ministry of Health (FMOH) also launched the tobacco-free, and physical activity initiative on April 17, 2014 [19]. In spite of this effort, some studies are elucidating the growing prevalence of abdominal obesity in Ethiopia [20-25].

The evidence of certain documented data elucidates abdominal obesity has been on the rise in Ethiopia, especially in urban areas. This could point to a failure to identify the elements that contribute to the development of abdominal obesity. As a result, we aimed to examine the prevalence and risk factors of abdominal obesity among urban adults in Jimma, Southwest Ethiopia.

## Methods

**Study design and setting:** a community-based cross-sectional study was conducted from February 1^st^ to March 1^st^, 2019, among adults in Jimma town, Ethiopia. Jimma town is located 335km southwest of Addis Ababa, the capital city of Ethiopia. The town has an area of 44.86sq.km with an altitude of 1750-2000m above sea level [26]. Based on the administration office report, the town has 17 kebeles (the smallest administrative unit of government structure) and 36,333 total households. As per the 2017/18 Health Bureau report, the town has 6 public health institutions and 18 private clinics. All adults above 18 years old and permanent residents of Jimma town were considered as the source population. Pregnant women, seriously ill, and adults with body deformities around the abdomen were excluded from the study.

**Sample size and sampling procedure:** a single population proportion formula was employed to determine the sample size. Using the assumptions: magnitude of abdominal obesity (P) of 50%, 95% confidence level, 5% marginal error, design effect of 2 and 10% non-response the final sample size becomes 845. A multi-stage sampling technique was applied to select study participants. Primarily, from a total of 17 urban kebeles found in the town, 6 kebeles were selected using a simple random sampling method by considering the WHO 30% inclusion for prevalence studies [27]. At the second stage, proportionally allocated 806 households were selected using systematic random sampling from each selected kebeles. Finally, one eligible adult was selected from each household using simple random sampling, where there was more than one eligible individual.

**Data collection tool and measurements:** a pretested semi-structured interviewer-administered questionnaire was prepared in accordance with the WHO-STEPs instrument for chronic disease risk surveillance and the Global Physical Activity Questionnaire (GPAQ) Analysis Guide and Guidelines for Measuring Household and Individual Dietary Diversity [28,29]. The questionnaire took account of socio-demographic information, dietary intakes, physical activity, and health risky behavior questions and anthropometric measurement was used.

The questionnaire was first prepared in English and translated to the local languages Amharic and Afan-Oromo then it was back-translated to English by translators blind to the original questionnaire to check the consistency. The questionnaire was pre-tested on 42 (5%) of the study population in Agaro town. A two-day training was given for the data collectors before the commencement of the data collection. Six diploma-level nurses with previous data collection experience were involved in the data collection. It was also supervised by two health officers.

Waist circumference was measured at a standing position midway between the lower rib margin and the anterior superior iliac crest in the horizontal plane using a flexible plastic metric tape to the nearest 0.1 cm. The measurement was taken when the participants were at the end of the gentle expiration, after taking a deep inhalation with the tape snug, but ensuring that it was not compressing the skin [28]. Abdominal obesity is defined based on the International Diabetic Federation (IDF) as waist circumference ≥94 cm for males and ≥80 cm for females [30].

The Food frequency questionnaire (FFQ) was used to assess the dietary habits of the participants included eight food categories (Meat, Egg, Fish, Fat rich food, Vegetables, Fruits, Dairy products, Sweet food), and was designed to obtain qualitative information about the usual food consumption patterns with an aim to assess the frequency with which certain food items or groups are consumed during a specific period. Participants were asked to report the frequency of a typical weekly consumption of each food group. A point was awarded to each food group consumed over the reference period, and the sums of all points were calculated for their individual dietary diversity score (DDS). The classification of DDS was obtained from the 8 food groups recommended by FAO. A scale was established for this distribution: low (1-4), and high (5-8) [31].

The Global Physical Activity Questionnaire (GPAQ) developed by the WHO was used to assess the physical activity pattern. Individuals were assessed by three domains: activities at work and travel, recreational activities, and sedentary behaviors. A vigorous-intensity activity was defined as any activity that causes a large increase in breathing or heart rate (e.g. running, carrying, or lifting heavy loads, digging, or construction work) that continued for at least 30 minutes for a minimum of three days per week. The moderate-intensity activity was defined as any activity that causes a small increase in breathing or heart rate (brisk walking or carrying light loads) that continued for at least 30 min for at least three days per week or five or more days of these activities for at least 20 min per day or more than 3 days of vigorous-intensity activity per week of at least 20 min per day. Low-level (sedentary) physical activity was defined as an individual having a physical activity that is not meeting any of the abovementioned criteria. Finally, the vigorous and moderate-intensity activities were categorized as physically active and sedentary physical activity as physically inactive [29].

Alcohol consumption was determined using Alcohol Use Disorders Identification Test (AUDIT) [32]. The AUDIT has 10 questions, each of the questions has a set of responses to choose from, and each response has a score ranging from 0 to 4. Total scores of 8 or more were considered as alcohol consumers and less than 8 were non-consumers. Alcohol was considered as intake of any type of locally prepared beverages or standard alcohol such as beer, and wine.

Current khat/chat (Catha Edulis) chewers were considered as if they had been chewing chat for more than 6 months and chew chat within 30 days preceding the study. Those who have never chewed khat are considered non-chewers. Smokers were defined as one who smoked at least 1 cigarette per day for a week those who smoked less than 1 cigarette per day or 7 cigarettes per week. Those who had never smoked at all were designated as non-smokers. Snack consumers were defined as eating any kind of food taken additionally between the three main meals irrespective of the size.

**Data analysis:** the data were checked, coded, cleaned, and entered using Epi-Data version 3.1 and exported to STATA version 14 for analysis. Simple linear regression was done to assess the association between dependent and independent variables. All variables having a p-value <0.25 in the simple linear regression were further entered into the multivariable linear model to control confounding effects. Basically, the linearity assumption was verified using both a scatter plot and a correlation matrix, and it was shown to be valid. The assumption of normality was confirmed using P-P plots, as well as by histogram with normality test. The homoscedasticity assumption was met by showing a scatter plot of standardized residuals versus standardized expected values, which was randomly distributed. The Durbin-Watson statistics were used to test the assumption of error and autocorrelation independence. The Durbin-Watson statistics for this data were 1.97, which is within the allowed range of 1.50 to 2.50, indicating that this study met the independence and autocorrelation assumptions. Multicollinearity was also tested by determining whether the standard error was less than 2, the Variance Inflation Factor (VIF) was less than 10, and the tolerance was greater than 0.1. As a result, no indication of multicollinearity was found.

After affirmation of the assumptions wealth index was generated using principal component analysis. The scores of 18 selected groups of assets and utilities were adapted from Ethiopian Demographic Health Survey (EDHS) and translated into latent factors. Bartlett's test of sphericity was checked and taken with p<0.05 as significant. Sampling adequacy for PCA was checked with Kaiser-Meyer-Olkin (KMO) and the measurement was accepted if it was >0.5. Varimax rotation is employed during factor extraction to minimize cross-loading of items on many factors. The factor that explained most of the variation was used to group study subjects into three ranked wealth groups.

**Ethical approval:** it was obtained from the Ethical Review Board of the Institute of Health, Jimma University. The necessary permission was obtained from the Jimma Town Health Office, administrative office, and from the selected kebele offices. Data were collected in line with the national ethical guidelines. Informed verbal consent was obtained from all participants before the interview and physical measurement. Participants were assured that they had the full right to participate or withdraw at any time during the study. Confidentiality of the information was assured by all the data collectors and principal investigators side. Brief advice was given for abdominally obese individuals after the data collection.

## Results

**Socio-demographic characteristics of study participants:** a total of 806 respondents participated in this study, making a response rate of 95.4%. Among the respondents 440 (54.6%) were females and 366 (45.4%) were males. The median age (IQR) of the study population was 30 (±15) years (Table 1).

**Table 1:** socio-demographic characteristics of study participants in Jimma town, Southwest Ethiopia, 2019

Variables		Frequency	Percentage
**Sex**	Female	440	54.6
	Male	366	45.4
**Age in year**	18-24	154	19.1
	25-34	370	45.9
	35-44	136	16.9
	45-54	104	12.9
	≥55	42	5.2
**Ethnicity**	Oromo	380	47.1
	Amhara	164	20.3
	South	244	30.3
	Tigre	18	2.2
**Educational status**	No formal education	38	4.7
	Primary level	184	22.8
	Secondary level	222	27.5
	Tertiary level	362	44.9
**Marital status**	Married	418	51.9
	Single	292	36.2
	Divorced	54	6.7
	Widowed	42	5.2
**Religion**	Orthodox	328	40.7
	Muslim	260	32.3
	Protestant	188	23.3
	Others	30	3.7
**Occupation**	Government employee	312	38.7
	Self- employee	272	33.7
	Student	74	9.2
	Unemployed	148	18.4
**Wealth index**	Poor	264	32.8
	Middle	274	34
	Rich	268	33.3
**Household size**	≤5 members	676	83.9
	>5 members	130	16.1

**Behavioural and dietary practices of the respondents:** concerning behavioural risk factors, about 289 (35.9%) of the participants consume alcohol. Most of the participants 684(84.9%) did not smoke a cigarette. About three hundred thirty-six (41.7%) of the respondents were chat chewers. Concerning physical activity, more than two-thirds (76.6%) of the participants were physically active. Regarding the participants' dietary characteristics, more than three fourth (83.5%) of participants consumed diversified food. About more than half (57.1%) of the participants did not have a snack, while half of the participants (52.8%) didn't skip breakfast (Table 2).

**Table 2:** behavioral and dietary characteristics of urban adults in Jimma town, Southwest Ethiopia, 2019

Variables		Frequency (N)	Percent (%)
**Alcohol drinking status**	No	517	64.14
	Yes	289	35.86
**Cigarette smoking**	No	684	84.86
	Yes	122	15.14
**Khat (Catha edulis) chewing**	No	470	58.31
	Yes	336	41.69
**Physical activity status**	Inactive	189	23.45
	Active	617	76.55
**Dietary diversity score**	Low	133	16.50
	High	673	83.50
**Snacking**	No	460	57.07
	Yes	346	42.93
**Breakfast skipping per week**	Didn't skip	426	52.85
	Once	92	11.41
	Twice	142	17.62
	Three and above	146	18.11

**Food consumption pattern of the respondents:** around 547(67.87%) of the respondents had vegetables 3-6 days per week. Nearly half of the participants (44.67%) had protein from animal source food 1-2 days per week. Two third 973.45%) of the respondents had consumed fats and oils source of food 3-6 days per week (Table 3).

**Table 3:** frequency of consumption of eight food groups among urban adults in Jimma town, Southwest Ethiopia, 2019

Variables		Frequency (N)	Percent (%)
**Vegetable**	None	15	1.86
	1-2 days/week	244	30.27
	3-6 days/week	547	67.87
**Fruits**	None	49	6.08
	1-2 days/week	375	46.53
	3-6 days/week	382	47.39
**Protein from animal source**	None	210	26.05
	1-2 days/week	360	44.67
	3-6 days/week	236	29.28
**Legumes**	None	16	1.99
	1-2 days/week	141	17.49
	3-6 days/week	649	80.52
**Dairy /milk**	None	358	44.42
	1-2 days/week	289	35.86
	3-6 days/week	159	19.73
**Cereals**	None	9	1.12
	1-2 days/week	260	32.26
	3-6 days/week	537	66.63
**Fats and oils**	None	4	0.50
	1-2 days/week	210	26.05
	3-6 days/week	592	73.45
**Sweets and sugars**	None	261	32.38
	1-2 days/week	321	39.83
	3-6 days/week	224	27.79

**Figure 1:**
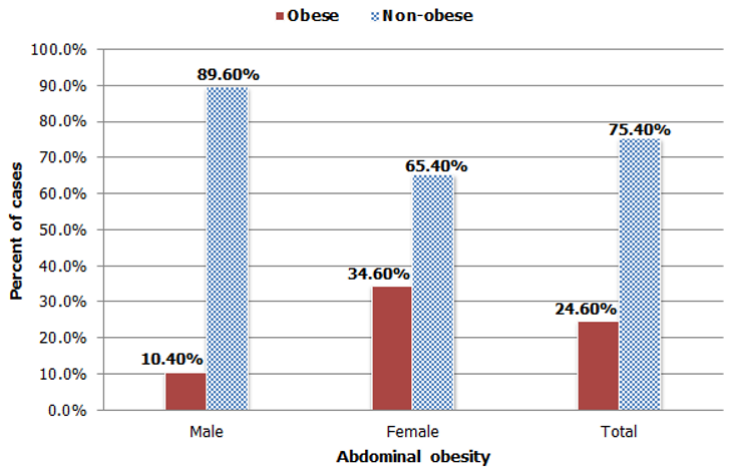
prevalence of abdominal obesity among adults in Jimma town, Southwest Ethiopia, 2019

**Prevalence of abdominal obesity:** the prevalence of abdominal obesity was 24.6% [95% CI: (21.5, 27.5)] and from the total magnitude, females constituted 16.3% (Figure 1).

**Factors associated with abdominal obesity:** variables including age, sex, alcohol consumption, smoking, chat chewing, dietary diversity, consuming snacks, household size, breakfast skipping, being sedentary, vegetable consumption, and physical inactivity were candidate variables for multivariable linear regression analysis. By kipping the other variables constant age, alcohol consumption, and physical inactivity were significantly associated with abdominal obesity among urban adults in Jimma town. The multivariable analysis model explained 71.8% of the variance in abdominal obesity (Adjusted R square = 0.718, P-value < 0.001). As the respondent's age increases by one-year, abdominal obesity increases by 0.32 (β= 0.319; 95%CI: 0.250, 0.389). When adults become alcohol consumers the risk of being abdominally obese increases by 1.63 (β= 1.631; 95%CI: 0.176, 3.087) as compared to non-alcohol consumers. As adults in this study become physically active the risk of being abdominally obese decreases by -2.05 (β= -2.053; 95%CI: -3.353, -0.454) as compared to physically inactive individuals (Table 4).

**Table 4:** multivariable regression model predicting abdominal obesity among urban adults in Jimma town, Southwest Ethiopia, 2019

Variables	Unstandardized Coefficients		P-value	95% Confidence Interval for β	
	β	Standard Error		Lower Bound	Upper Bound
**Sex**	-1.183	.844	0.162	-2.840	.475
**Age**	.319	.035	0.000*	.250	.389
**Smoking**	.277	1.080	0.797	-1.843	2.398
**Alcohol consumption**	1.631	.741	0.028*	.176	3.087
**Chat chewing**	-.779	.827	0.346	-2.402	.844
**Dietary diversity score**	.175	.928	0.850	-1.646	1.996
**Breakfast skipping**	-.082	.287	0.776	-.644	.481
**Sedentary**	.003	.002	0.243	-.002	0.007
**Snacking**	.683	.687	.0.320	-.665	2.030
**Physically activity**	-2.053	.815	0.012*	-3.653	-0.454
**Household size**	1.515	.929	0.104	-.310	3.339
**Vegetable consumption**	.216	.183	0.238	-.143	0.575

β**=**Regression coefficient, *=Significant association

## Discussion

Obesity is rapidly reaching epidemic proportions worldwide and has been associated with numerous co-morbidities and an increased risk of premature death. Independent of general obesity, abdominal deposition of adipose tissue is directly related to cardiovascular morbidity and mortality [10,12]. It has also been suggested that abdominal obesity is the key determinant for the increasing prevalence of metabolic syndrome in sub-Saharan countries including Ethiopia [33]. The non-communicable diseases burden has cost the developing countries both economic and human capital. Therefore, identifying the factors associated with obesity especially the easily liable fat on abdominal obesity is a basic cost-effective prevention for halting the NCDs effect. In this study, the overall magnitude of abdominal obesity was 24.6%. Age, alcohol use, and physical inactivity were significantly associated with abdominal obesity. The finding of this study strengthens reports from previous studies that obesity is becoming a silent epidemic in the country and calling policymakers for timely intervention [5,6,20].

The prevalence of abdominal obesity was 24.6 % in this study, which is consistent with studies conducted in Combolcha town, Dilla town, and Nekemte town [22-24]. However, the finding was lower than studies conducted in Dire Dawa administrative city, and Northwest Ethiopian [25,34]. On the other hand, our finding is higher than the results of studies done in Addis Ababa, Bona District, Mizan-Aman town, Woldia town [20,21,35,36], and Benin [37]. The observed difference may be due to the socio-demographic and ethnic background differences of the study participants [38].

Besides, the residence of the participants might also be another possible reason for the discrepancy in the study findings. For instance, individuals who live in urban areas and developed countries are at higher risk of getting abdominal obesity as compared to rural residents due to their more frequent overconsumption of energy-dense and processed foods [38,39]. As an individual consumes high energy-containing diet, the metabolic activity pathway shifts into an anabolic process, this, in turn, leads to an increment of fats in the circulatory system and the need to store them to maintain the metabolic activities. When the energy intake exceeds expenditure, the excess fat will be accumulated in adipose tissue unlimitedly and finally will result in those individuals becoming abdominally obese [40]. In addition, the difference in cutoff point used for waist circumference in the studies might be another possible justification for the discrepancies. This is because the cutoff points to determine abdominal obesity across different ethnicities are slightly different due to the composition and shape of body differences between diverse ethnicities [41]. The methods that are used to identify abdominal obesity may also be the other reason.

Regarding factors associated with abdominal obesity, the risk of becoming abdominally obese increases as the age increases. The findings of the current study were supported by studies done in Dire Dawa city administration, Nekemte town, urban areas of Northwest Ethiopia [24,25,34], and Northern Iran [42]. This could be because when people are getting old they start to decrease their physical activity level, which will result in a positive energy balance and leads to the development of abdominal obesity. In addition, studies have reported that being young is a statistically protective factor for the development of abdominal obesity [43]. This might be due to the fact that the basal metabolic activity of the body gradually declines as we get older. Low metabolic activity leads to a decrease in energy expenditure and more storage of fat in the body even with a low intake diet. In addition, as individuals get older, the fat distribution and accumulation shift into the abdominal region, and will have a chance to develop abdominal obesity [44].

This study found that physically inactive participants had a higher chance of becoming abdominally obese as compared to physically active individuals. This finding was supported by a study done in Dilla town, Dire Dawa city administration [23,25], and Ghana [45]. The lower risk of being abdominally obese among physically active participants might be due to physical activity having a role in altering the balance between energy intake and energy expenditure. For instance, when an individual is physically active or has regular exercise the body energy expenditure will exceed the energy intake and the stored fat will be utilized. It was evidenced that during exercise fat from the abdominal regions of the body will be utilized preferentially [46]. Furthermore, the cultural misconception regarding abdominal obesity that is found in most parts of African countries could be the reason behind the increased odds of being abdominally obese. To illustrate, individuals associate being abdominally obese with prestige, happiness, and a good healthy living lifestyle [47].

The risk of becoming abdominally obese was 1.6 times more likely among individuals who were alcohol drinkers than non-drinkers. This finding was supported by studies conducted in Dire Dawa city administration [25], and Ghana [48]. This might be due to that alcohol is an extra dense energy that adds to the total daily energy intake and allows the macronutrients to be stored without substituting the food [49]. In addition, alcohol inhibit fat oxidation due to the anti-lipolytic properties of metabolites from alcohol degradation [50]. Subsequently, this will favor the storage of lipid in adipose tissue and hence promotes an increased risk of developing abdominal obesity.

The strength of our study was that it is a large population-based study consisting of 845 Ethiopian men and women. Principal component analysis (PCA) was used for the wealth index analyses. However, there could be differences in the magnitude of abdominal obesity by season, which this study was not able to assess. Because of the slender body frame of Ethiopians, the cutoff used for abdominal obesity may underreport the prevalence. The study was also unable to do advanced methods of measuring abdominal adiposity. The women and even some males might not self-report their alcohol-drinking and smoking habits which are socially undesirable behaviors within the community. The cross-sectional nature of the study design is also another limitation.

## Conclusion

Compared to different previous studies, the magnitude of abdominal obesity among adults in the study area was high. Age, alcohol use, and physical inactivity showed an association with the observed obesity among the adult population of Jimma Town. Therefore, the established sports commission-driven promotion to increase the communities' level of physical activity should be strengthened. Apart from the government tax increment sanction on alcohol industries, the regular media and social media should promote the community to reduce the level of alcohol intake. Finally, further longitudinal research is needed to identify factors that can be altered by seasonality.

### What is known about this topic

- *Obesity once the problem of the developed countries is becoming the issue of developing countries, resulting in double burden;*
- *Behavioral factors and sedentary life styles are responsible for the development of abdominal obesity*.

### What this study adds

- *The result finds out the high magnitude of abdominal obesity in the particular study area;*
- *The study has also identified modifiable factors that have impact on the development of abdominal obesity in the study area*.
